# Differential Expression of Immune Checkpoint Modulators on *In Vitro* Primed CD4^+^ and CD8^+^ T Cells

**DOI:** 10.3389/fimmu.2016.00221

**Published:** 2016-06-16

**Authors:** Nina C. Sabins, Benjamin C. Harman, Linda R. Barone, Shixue Shen, Sandra Santulli-Marotto

**Affiliations:** ^1^Biologics Research, Janssen R&D, Spring House, PA, USA

**Keywords:** PD-1, TIM-3, immune checkpoint, T cells, *in vitro*

## Abstract

PD-1, TIM-3, and LAG-3 are molecules shown to have immune modulatory properties, and although initially classified as indicators of T cell hyporesponsiveness, it has become clear that they are also associated with the normal course of T cell activation. Functional studies have focused mainly on CD8^+^ T cells during chronic inflammation due to interest in co-opting the cellular immune response to eliminate viral or cancerous threats; however, there remains a relative lack of data regarding the expression of these molecules on CD4^+^ T cells. Here, we report that expression of the immune checkpoint (IC) molecules PD-1, LAG-3, and TIM-3 are differentially expressed on CD4^+^ and CD8^+^ T cells in the allogeneic response resulting from a mixed lymphocyte reaction. In these studies, PD-1 expression is higher on CD4^+^ T cells compared to CD8^+^ T cells. In contrast, TIM-3 is expressed at higher levels on CD8^+^ T cells compared to CD4^+^ T cells with an apparent reciprocity in that PD-1^+^ CD4^+^ T cells are frequently TIM-3^lo/−^, while TIM-3-expressing CD8^+^ T cells are largely PD-1^lo/−^. In addition, there is a decrease in the frequency of TIM-3^+^ CD4^+^ cells producing IFN-γ and IL-5 compared to TIM-3^+^ CD8^+^ cells. Lastly, the memory T cell phenotype within each IC-expressing subset differs between CD4^+^ and CD8^+^ T cells. These findings highlight key differences in IC expression patterns between CD4^+^ and CD8^+^ T cells and may allow for more effective therapeutic targeting of these molecules in the future.

## Introduction

T cells exposed to chronic antigen stimulation, particularly in the context of viral infection or the tumor microenvironment, are characterized as exhausted upon loss of proliferative potential, cytokine production, and cytotoxicity (1). Accompanied by progressive loss of function, these cells also acquire an altered phenotype with accumulated expression of what have been classified as immune checkpoint (IC) molecules, such as PD-1, TIM-3, and LAG-3. Ligand binding to PD-1 and TIM-3 has been shown to exacerbate T cell dysfunction. In the context of allogeneic transplantation, overexpression or agonism of these signaling pathways led to allograft tolerance, whereas absence or blockade led to accelerated rejection and lethality in mice (2–4). Several studies have shown that blocking IC interactions restored and/or enhanced T cell effector function (5–7). However, it is also known that CD4^+^ and CD8^+^ T cells expressing multiple IC during a normal response are not necessarily functionally compromised, but rather represent a phase of T cell activation (8), and that these molecules serve a costimulatory role in T cell activation (9–12). In addition, expression of PD-1, TIM-3, and LAG-3 has been associated with activation and differentiation (13, 14), with reports suggesting that certain differentiation stages, effector memory (EM) in particular, correlate with increased function (10, 15).

Immune checkpoint-expressing CD8^+^ T cells isolated from HIV-infected patients can be associated with both increased or decreased cytotoxicity and viral load (12, 16, 17). In cancer, several groups have shown that IC blockade can restore antitumor immunity in T cells (7, 18), providing justification for targeting these molecules in the clinic (19, 20). However, it has also been reported that high expression of PD-1 on CD8^+^ tumor infiltrating lymphocytes correlates with antitumor specificity and reactivity (21). These paradoxical findings indicate that the context in which IC expression is analyzed can lead to variable predictions of T cell function. Previous *in vitro* studies analyzing IC expression have implemented CD3/CD28 cross-linking for T cell activation (13), which, while informative, excludes the impact of IC ligands and soluble factors from viable antigen presenting cells. In addition, extensive studies have focused on IC expression and function of CD8^+^ T cells with less known regarding IC expression on CD4^+^ T cells; and while CD8^+^ T cells are major drivers of viral and tumor clearance, CD4^+^ T cell help plays a major role in these responses. An analysis of IC expression on both CD4^+^ and CD8^+^ T cell expression could help optimize therapeutic IC blockade (or agonism). Here, we employ a modification of the mixed lymphocyte reaction (MLR) to dissect the differences in IC expression levels and kinetics on CD4^+^ and CD8^+^ T cells to define expression patterns during a physiological immune response. We report that expression of PD-1, LAG-3, and TIM-3 coincides with T cell activation and function, but these molecules are differentially expressed on CD4^+^ and CD8^+^ T cells. In addition, CD4^+^ T cells undergoing proliferation that express PD-1 often exhibit lower expression of TIM-3, while TIM-3 expressing CD8^+^ T cells have reduced PD-1 expression. These differences extend to cytokine production in that IC expression differs between cytokine-producing CD4^+^ and CD8^+^ T cells. Lastly, we find that CD4^+^ and CD8^+^ T cells exhibit different memory T cell phenotypes depending on which of these molecules are expressed.

## Materials and Methods

### Primary Cells

Purified human pan T cells from healthy donors were purchased from Biological Specialty Corporation (Colmar, PA, USA). T cells were confirmed to be >95% CD3^+^ by flow cytometry. Human monocyte-derived dendritic cells (DCs) from healthy donors were purchased from Astarte Biologics (Bothell, WA, USA) and confirmed to be >90% CD11c^+^, and >90% CD83^+^, CD86^+^, and HLA-DR^+^ after activation.

### Mixed Lymphocyte Reaction

T cells and DCs were cultured in complete media consisting of RPMI 1640 with Glutamax (Life Technologies, Grand Island, NY, USA), supplemented with 5% heat inactivated human serum (Sigma, St. Louis, MO, USA). DC were cultured overnight with 500 U/mL each of recombinant IL-4 and GM-CSF (Peprotech, Rocky Hill, NJ, USA) and matured with 1000 U/mL recombinant IFN-γ (Peprotech) and 1 ng/mL LPS (Sigma). Prior to coculture with DC were tested for maturation status by CD83, CD86, and HLA-DR expression by flow cytometry and IL-12 production by ELISA (R&D Systems, Minneapolis, MN, USA). T cells were labeled with violet proliferation dye 450 (BD) according to the manufacturer’s instructions. T cells were cultured with DC at a 10:1 ratio, incubated at 37°C for the indicated timepoints, and analyzed for proliferation and activation by flow cytometry. Supernatants were collected and cytokines were measured by multiplex analyses (MesoScale Discovery, Rockville, MD, USA). For ELISPOT analysis, cells were collected on day 6 of MLR and analyzed for IFN-γ spot production using pre-coated plates (MabTech, Cincinnati, OH, USA). For intracellular detection of cytokines, cells were collected on day 6 of the MLR and treated with PMA (Sigma), ionomycin (Sigma), and GolgiPlug (BD, San Jose, CA, USA) for 6 h prior to addition of antibodies for flow analysis.

### Flow Cytometry

All cells were labeled with live/dead dye near infra red (Life Technologies) for dead cell exclusion and treated with Fc Block (Miltenyi, San Diego, CA, USA) prior to staining with fluorescently labeled antibodies. Anti-human antibodies used for DC staining were anti-CD83 PE (Biolegend, San Diego, CA, USA), anti-CD86-PE-Cy7 (Biolegend), anti-HLA-DR V450 (BD), and anti-CD11c APC (BD). Antibodies used in the T cell characterization were anti-LAG-3 FITC (Novus, Littleton, CO, USA), anti-PD-1 PerCP-Cy5.5, anti-CD3 Alexa700, anti-CD4 Brilliant Violet 650, anti-CD8 Brilliant Violet 570, anti-IFN-γ PE, anti-IL-5, anti-CD62L PE, and anti-CD45RA Alexa700 (all, Biolegend), anti-CD25 PE, and anti-IFN-γ PE-Cy7, anti-IL-4 PE-Cy7 (all BD), and anti-TIM-3 APC (R&D Systems). Surface staining and intracellular staining was performed using Cytofix/Cytoperm™ Plus kit (BD) according to the manufacturer’s instructions. Data acquisition was performed using the LSRFortessa flow cytometer (BD). Data analysis was performed with FlowJo version 9 (Tree Star, Inc., Ashland, OR, USA).

### Quantification and Statistical Analysis

For all markers analyzed by flow cytometry, isotype controls were used to establish gates by setting gates between 0.5 and 1% positive events. Gates were drawn around each cell division as determined by violet proliferation dye dilution, and subsequent populations were analyzed for expression of CD25, PD-1, LAG-3, and TIM-3. Quantifications were made based on data generated from FlowJo, and statistical analysis was performed using GraphPad Prism version 6. Statistical significance was determined using two-tailed paired *t*-test (Holm–Sidak) and two-way ANOVA (Dunnet’s). Correlation coefficients were determined using two-tailed paired monotonic analysis (Spearman).

## Results

### Differential Kinetics of Immune Checkpoint Molecule Expression on Primed T Cell Populations

**Figure 1:**
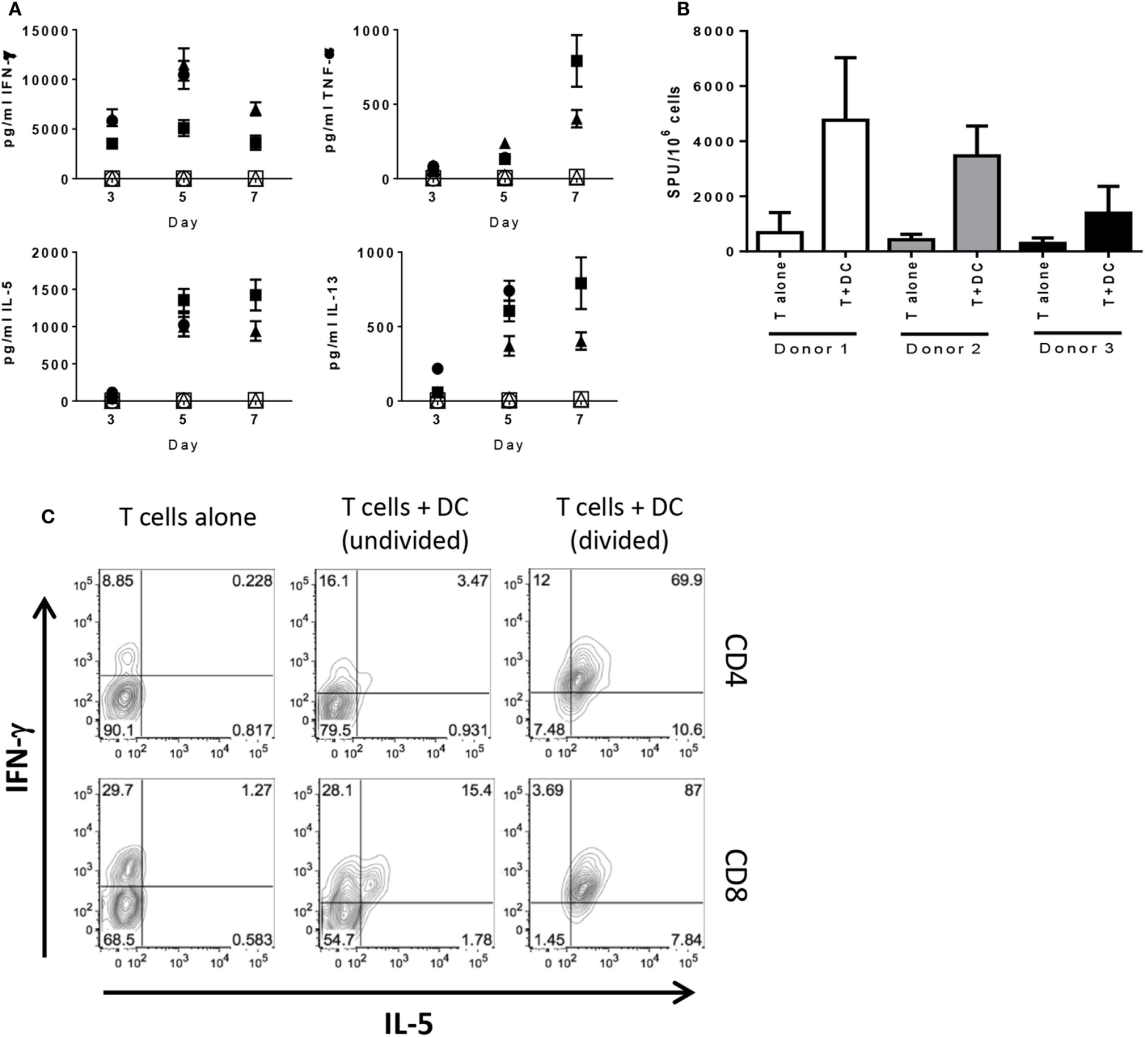
**T cells stimulated by allogeneic DC do not exhibit Th1/Th2 bias in cytokine production**. Human pan T cells from multiple donors were cultured *in vitro* with allogeneic DC that had been activated with LPS and IFN-γ. **(A)** Time-course analysis by MSD of cytokine levels accumulated in MLR supernatants; panels clockwise from the left: IFN-γ, TNF-α, IL-5, and IL-13; filled shapes represent T cells cultured with DC, open shapes represent T cells cultured alone (three donors). Cells collected from day 6 of MLR were analyzed for **(B)** IFN-γ spot forming units by ELISPOT or **(C)** CD4^+^ and CD8^+^ production of IFN-γ and IL-5 by flow cytometry. Contour plots representative of three independent experiments after fluorescence compensation and gating on live cells → singlets → CD3^+^ T cells → CD4^+^ versus CD8^+^ T cells.

To analyze T cell activation and kinetics of IC expression in an acute primary response, purified pan T cells from four different donors were cultured with allogeneic DC and analyzed at various time points. We chose to mature the DC prior to addition to MLR to maximally activate the T cells. As mentioned in Section “Materials and Methods,” we confirmed activation by upregulation of CD83, CD86, and HLA-DR, as well as production of IL-12p40 and IL-12p70 (Figures S1A,B in Supplementary Material). T cells were cultured with DC and gated on CD4^+^ and CD8^+^ T cells for analysis. As expected, both CD4^+^ and CD8^+^ T cells exhibited progressively increasing proliferation (Figure S1C in Supplementary Material) with the majority of T cells divided after 7 days and minimal proliferation by T cells cultured alone. Longitudinal analyses of supernatants collected from these cultures showed levels of TNF-α, IL-5, and IL-13 increased over time with steady levels of IFN-γ up to day 7 of MLR, while T cells alone did not produce detectable levels of cytokine (Figure 1A), as was the case for DC cultured alone (data not shown). Subsequent analyses of the T cells were performed on day 6 of MLR given that proliferation and cytokine production were measureable and still increasing, thus capturing T cells approaching peak activation. ELISPOT analysis showed cells actively producing IFN-γ when cultured with allogeneic DC, whereas T cells alone exhibited little to no spots (Figure 1B). We wanted to confirm these findings by intracellular staining for cytokine. A population of CD4^+^ and CD8^+^ T cells that produced IFN-γ, but not IL-5, was observed when cultured in absence of DCs, presumably in response to the PMA/ionomycin stimulation. In the undivided population, there was a subset observed in both CD4^+^ and CD8^+^ T cells that produced IFN-γ or IFN-γ and IL-5 together with very few producing IL-5 alone. The dual-cytokine-producing population was markedly increased on the divided population for both CD4^+^ and CD8^+^ T cells (Figure 1C). Together, these data demonstrate that the T cells are activated and likely the main source of cytokine measured in the supernatant of the MLR, thus showing that both CD4^+^ and CD8^+^ T cells are primed and functional after 6 days of stimulation by allogeneic DC. Additionally, we show that, although the allogeneic DC used as stimulators were activated with LPS and IFN-γ and produced IL-12, they did not polarize the T cells toward Type 1 or Type 2, given that both Th1 and Th2 cytokines were observed.

**Figure 2:**
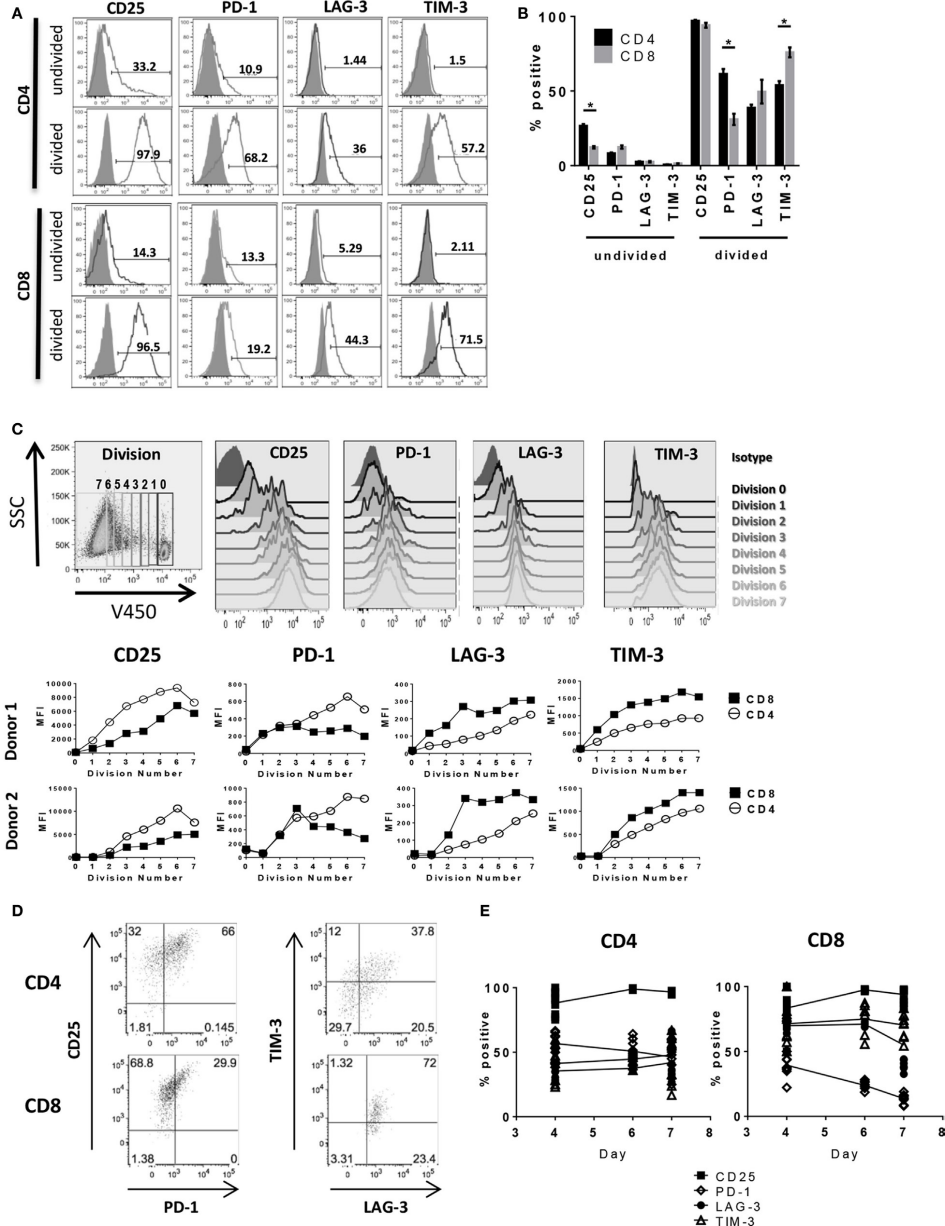
**Activated T cells express PD-1, LAG-3, and TIM-3**. Human pan T cells were stimulated with allogeneic DC for 6 days. **(A)** Representative flow cytometry histograms of CD4^+^ and CD8^+^ T cells gated on the responding (divided) or non-responding (undivided) population based on proliferation dye dilution, and analyzed for CD25, PD-1, LAG-3, and TIM-3 expression (black lines) compared to isotype (shaded gray). **(B)** Graphical representation of IC expression on non-responding (undivided) and responding (divided) CD4^+^ (black bars) and CD8^+^ T cells (gray bars). Data pooled from four independent experiments. *p < 0.0001, two-way ANOVA (Dunnet’s). **(C)** Representative gating strategy of CD25, PD-1, LAG-3, and TIM-3 expression within each cell division (top panels). Graphical analyses depicting expression of each IC for CD4^+^ (open circles) and CD8^+^ (closed circles) T cells by geometric MFI for two donors (bottom panels). **(D)** Representative dot plots of co-expression of CD25, PD-1, TIM-3, and LAG-3 on CD4^+^ and CD8^+^ T cells. Plots of TIM-3 versus LAG-3 were drawn from CD25^+^ PD-1^+^ gates. Data are representative of three independent experiments. **(E)** Trendlines depicting expression of CD25 (filled squares), PD-1 (open diamonds), LAG-3 (filled circles), and TIM-3 (open triangles) on dividing CD4^+^ and CD8^+^ T cells over time. Data representative of three different donors at each time point compiled from two independent experiments.

We next performed a detailed kinetics study of IC expression on activated T cells by flow cytometry (complete gating strategy shown in Figure S2 in Supplementary Material). By 6 days after stimulation, nearly all dividing CD4^+^ and CD8^+^ cells expressed activation marker CD25 (>95%), whereas the non-dividing CD4^+^ and CD8^+^ populations had a much lower frequency of CD25^+^ cells (26.6 ± 2.2 and 12.3 ± 1.2%, respectively) and lower overall mean fluorescence intensity (MFI) as shown in Figure 2 and Table 1. The frequency of CD25^+^ CD4^+^ T cells was significantly higher than that of CD25^+^ CD8^+^ (Figures 2A,B; Table 1); however, due to lack of proliferation and expression of other IC markers, these cells are unlikely to be responders. PD-1 expression was detectable in a small population of non-dividing cells (<15%), with even less or no LAG-3 and TIM-3 expressed in the population of non-dividing cells. Expression of PD-1, LAG-3, and TIM-3 was largely restricted to dividing cells, similar to CD25 (Figures 2A,B; Table 1), albeit at different levels between CD4^+^ and CD8^+^ T cells as well as within each population. On CD4^+^ T cells, PD-1 expression was higher (61.4 ± 6.2%) compared to CD8^+^ T cells (31.0 ± 7.2%). Conversely, CD4^+^ expression of TIM-3 was lower (53.9 ± 4.9%) compared to TIM-3 expression on CD8^+^ T cells (75.9 ± 6.2%). LAG-3 expression on CD4^+^ (38.8 ± 3.8%) was lower than PD-1 and TIM-3; however, LAG-3 expression on CD8^+^ (49.6 ± 15.2%) varied between donors with no significant trend observable. Expression levels of each IC on CD4^+^ and CD8^+^ T cells across four donors are summarized in Table 1. This heterogeneity of IC expression between CD4^+^ and CD8^+^ T cells was evident even though analysis was restricted to dividing cells that were CD25^+^ indicating all were activated. To better understand the relationship between cell division and expression of PD-1, LAG-3, and TIM-3 during T cell activation, further analysis of IC molecule expression was performed by gating on iterative population doublings based on proliferation dye dilution over the course of the 6-day culture. As shown in Figure 2C, both CD4^+^ and CD8^+^ T cells exhibited MFI of each IC molecule in correlation with the number of cell divisions, i.e., the more a T cell had divided, the higher the expression of PD-1, LAG-3, and TIM-3, coinciding with the same expression kinetics as CD25. Interestingly, the MFI of CD25 and PD-1 was consistently higher on CD4^+^ T cells compared to CD8^+^ T cells, whereas the MFI of LAG-3 and TIM-3 was higher on CD8^+^ T cells compared to CD4^+^. The broad distribution of IC expression observed on dividing CD4^+^ and CD8^+^ T cells prompted us to evaluate co-expression between these molecules. Upon closer examination of CD25^+^ proliferating cells, PD-1 presented with differential expression between CD4^+^ and CD8^+^ T cells. The majority of CD25-expressing CD4^+^ T cells also expressed PD-1 (66%), whereas of the CD25^+^ CD8^+^ T cells, only 30% expressed PD-1 (Figure 2D). Gating on CD25^+^ PD-1^+^ double positive CD4^+^ T cells, we found that 58% expressed LAG-3 and 37% expressed all four. CD25^+^ PD-1^+^ double positive CD8^+^ T cells had even higher expression of the other two IC, with ~95% expressing LAG-3 and ~75% expressing TIM-3 (Figure 2D). To determine the kinetics of expression of these markers throughout the priming phase, we performed longitudinal analyses of activated T cells in the MLR. Although by day 4, very few cells had divided (Figure S1C in Supplementary Material), we found that within the population of the dividing cells, nearly all expressed CD25 and that this expression was sustained through day 7 (Figure 2E). A subset of both dividing CD4^+^ and CD8^+^ cells upregulated TIM-3, LAG-3, and PD-1 at day 4; however, while we showed that the number of dividing cells increased over time, PD-1 expression began to decrease by day 6 with a more significant reduction observed on CD8^+^ T cells (Figure 2E). LAG-3 expression also decreased by day 7 on CD8^+^ T cells, but not on CD4^+^, while TIM-3 expression remained stable on both subsets (Figure 2E). These findings indicate that, within the context of an *in vitro* allogeneic response, PD-1, LAG-3, and TIM-3 expression coincide with T cell activation, but show that the expression kinetics differ between CD4^+^ and CD8^+^ T cells. Responding CD8^+^ T cells exhibited lower initial PD-1 expression compared to CD4^+^ T cells, with more rapid downregulation further highlighting the heterogeneity of IC expression in the responding CD4^+^ and CD8^+^ T cell populations.

**Table 1:** **Immune checkpoint expression on dividing and non-dividing CD4^+^/CD8^+^ T cells**.

	CD4	CD8
**Undivided (mean ± SEM)**		
CD25	28.45 ± 1.83	15.10 ± 1.84
	30.96 ± 1.25	12.76 ± 0.61
	26.37 ± 0.26	9.09 ± 0.37
	20.67 ± 0.12	12.20 ± 0.81
PD-1	5.48 ± 0.10	7.22 ± 0.08
	10.54 ± 0.19	12.40 ± 0.49
	7.92 ± 0.25	13.23 ± 0.84
	8.95 ± 0.41	16.90 ± 0.71
LAG-3	2.31 ± 0.15	1.21 ± 0.15
	1.06 ± 0.07	2.96 ± 0.74
	3.84 ± 0.08	1.78 ± 0.29
	3.42 ± 0.19	4.39 ± 0.61
TIM-3	1.01 ± 0.13	1.78 ± 0.23
	1.04 ± 0.14	1.54 ± 0.08
	0.72 ± 0.10	1.30 ± 0.28
	0.07 ± 0.06	1.14 ± 0.16
**Divided (mean ± SEM)**		
CD25	97.19 ± 0.15	97.44 ± 0.20
	97.68 ± 0.03	96.45 ± 0.15
	99.17 ± 0.48	97.47 ± 0.59
	94.43 ± 1.05	85.00 ± 2.02
PD-1	47.72 ± 1.52	21.22 ± 1.38
	59.46 ± 0.73	24.19 ± 1.28
	60.50 ± 2.14	26.13 ± 1.60
	78.03 ± 2.08	52.47 ± 0.45
LAG-3	30.89 ± 2.07	19.91 ± 0.42
	37.72 ± 1.73	27.12 ± 2.28
	37.53 ± 1.27	71.00 ± 1.16
	49.13 ± 1.03	80.20 ± 0.79
TIM-3	57.84 ± 2.44	80.12 ± 0.94
	61.63 ± 2.93	86.74 ± 0.59
	39.40 ± 1.88	58.03 ± 2.28
	56.57 ± 1.66	78.87 ± 1.30

*Human pan T cells were stimulated with allogeneic DC for 6 days. CD4^+^ and CD8^+^ T cells were analyzed by flow cytometry and gated on responding (divided) or non-responding (undivided) population based on proliferation dye dilution, and analyzed for CD25, PD-1, LAG-3, and TIM-3 expression. Mean and SEM were calculated from three biological replicates per donor. Data from four donors are shown*.

### Activated CD4^+^ and CD8^+^ T Cells Differ in Expression Patterns of Immune Checkpoint Molecules

**Figure 3:**
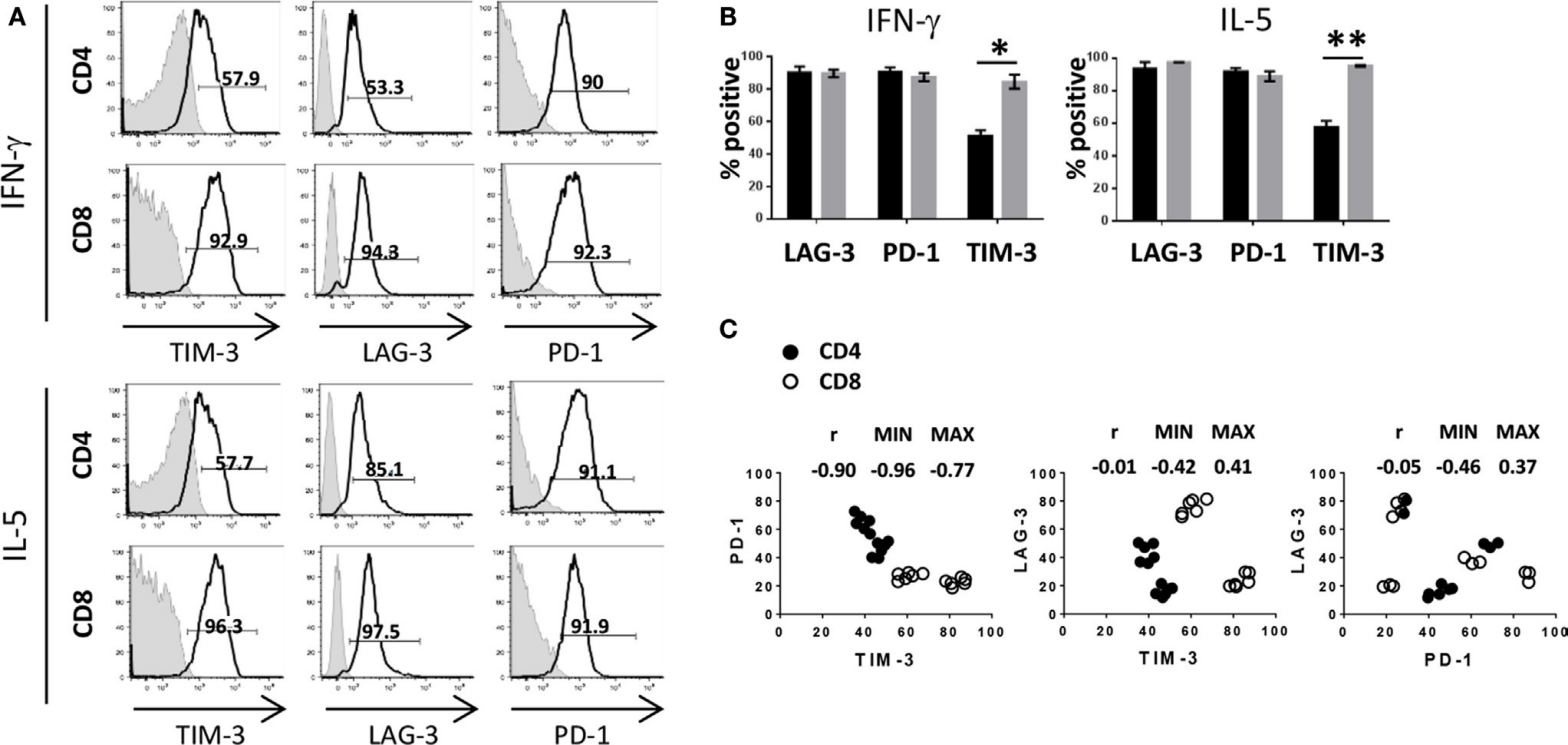
**Differential expression of immune checkpoints between CD4^+^ and CD8^+^ T cells**. Human pan T cells were stimulated with allogeneic DC for 6 days, as done in Figure 2, and analyzed for intracellular cytokines as done in Figure 1. **(A)** Gates were drawn on IFN-γ or IL-5-positive CD4^+^ and CD8^+^ T cells and expression of TIM-3, LAG-3, and PD-1 (black lines) is shown compared to isotype control (shaded gray) as percent positive events. **(B)** Graphical representation LAG-3, PD-1, and TIM-3 expression levels on IFN-γ and IL-5 producing CD4^+^ (black) and CD8^+^ (gray) T cells. Data are mean ± SEM compiled from three donors. *p < 0.005, **p < 0.001, two-tailed *t*-test (Holm–Sidak). **(C)** Expression of PD-1, LAG-3, and TIM-3 was analyzed on activated CD4^+^ (closed circles) or CD8^+^ (open circles) T cells. TIM-3 expression was compared to PD-1 (left panel), LAG-3 (center panel), and PD-1 expression was compared to LAG-3 (right panel). Spearman’s rank correlation coefficients *r*, along with 95% confidence intervals (MIN and MAX), are shown. Data compiled from four different donors conducted in three independent experiments.

Having shown that activated and proliferating T cells expressed IC to varying degrees as well as with differential kinetics, we were then interested in analyzing IC expression in association with cytokine production. Based on the cytokine milieu measured in the MLR (Figure 1A), we focused our subsequent analyses on IFN-γ and IL-5 and observed that cytokine production was associated with high expression of PD-1 and LAG-3 for both CD4^+^ and CD8^+^ T cells. However, expression of TIM-3 was significantly decreased on cytokine-producing CD4^+^ T cells compared to cytokine-producing CD8^+^ T cells (Figures 3A,B). As shown in the previous section, the frequency of TIM-3-expressing CD8^+^ T cells was consistently higher compared to the frequency of CD4^+^ T cells from each donor tested, whereas PD-1 appears to be expressed on CD4^+^ at a slightly higher frequency compared to CD8^+^ T cells. Figure 3C illustrates the relationship between expression patterns of each IC molecule on CD4^+^ and CD8^+^ T cells. A negative correlation was observed in PD-1 and TIM-3 expression, in which cells that expressed high PD-1 had lower TIM-3 expression and cells with low PD-1 and higher TIM-3 [Spearman test *r* value (*r*) in the pair comparison was 0.90, *p* < 0.0001]. Of note, PD-1^hi^TIM-3^lo^ expression was largely restricted to CD4^+^ T cells, whereas CD8^+^ T cells were predominantly PD-1^lo^TIM-3^hi^ (Figure 3C). No correlation was observable with LAG-3 and TIM-3 or PD-1 (*r* = −0.05, *p* = 0.81 and *r* = −0.01, *p* = 0.96, respectively). Taken together, these results show that although PD-1, TIM-3, and LAG-3 are upregulated on activated T cells, the expression pattern of these molecules, in particular PD-1 and TIM-3, differs between CD4^+^ and CD8^+^ T cells.

### Immune Checkpoint Positive Cells Exhibit Differential Memory Phenotype between CD4^+^ and CD8^+^ T Cells

**Figure 4:**
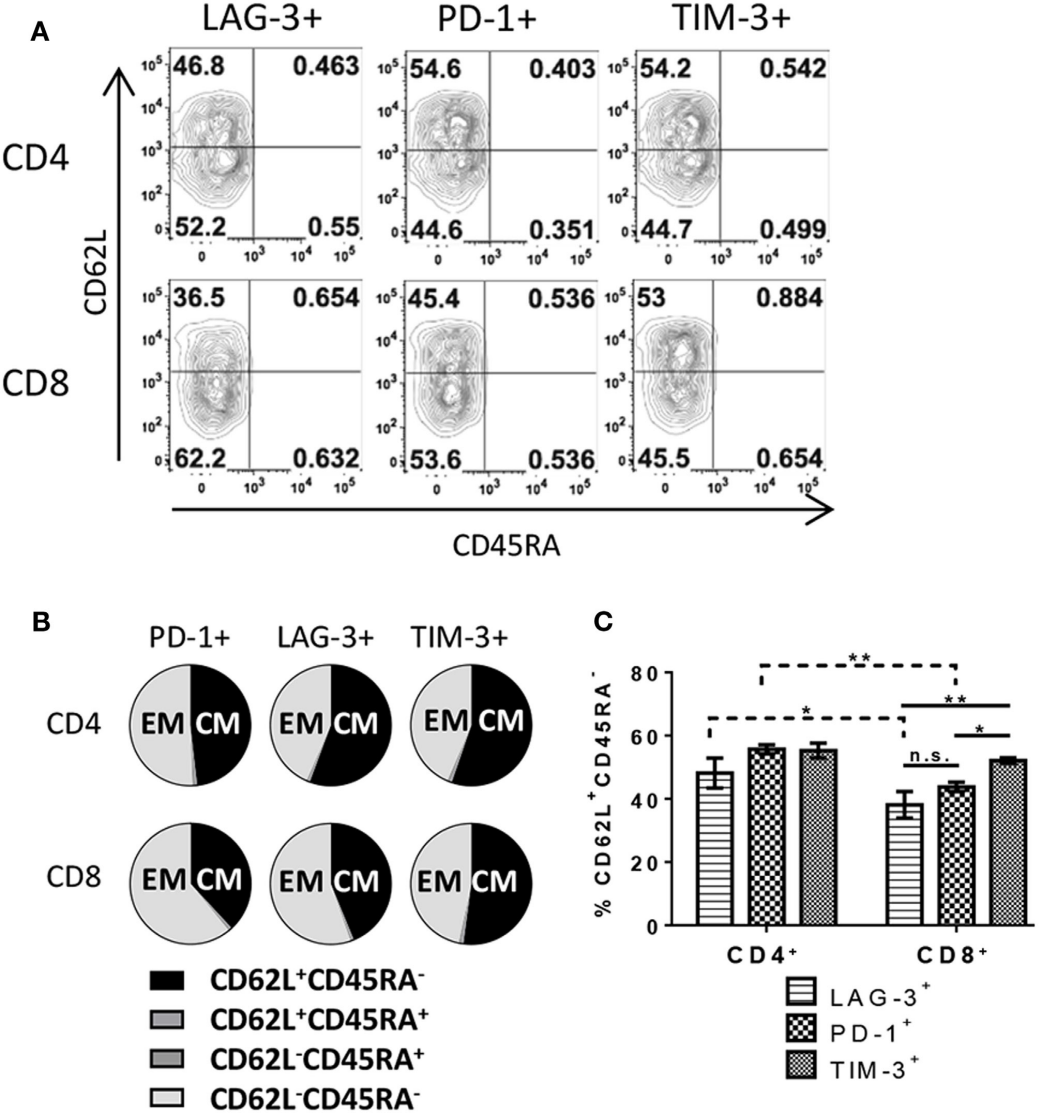
**T cells expressing immune checkpoint molecules exhibit central and effector memory phenotypes**. Human pan T cells were stimulated with allogeneic DC for 6 days, as done in Figure 2. LAG-3, PD-1, and TIM-3 expressing CD4^+^ and CD8^+^ T cells were subsequently analyzed for CD45RA and CD62L expression. **(A)** Representative flow cytometric contour plots, gated on dividing CD4^+^ or CD8^+^ T cells, depicting quadrant gates separating different T cell memory subsets. **(B)** Pie charts quantifying T cell memory subsets within each immune checkpoint molecule expressing population. **(C)** Bar graph comparing frequencies of CD62L^+^ CD45RA^−^ cells within each immune checkpoint molecule expressing population. Data are mean ± SEM compiled from three donors. *p < 0.05, **p < 0.01, two-way ANOVA (Dunnet’s).

There are several possible outcomes during T cell response to antigen including, but not limited to, differentiation into subsets with defined functions such as effector cells, EM cells, or central memory (CM) cells. To further understand the difference in IC expression between CD4^+^ and CD8^+^ T cells, each subset was analyzed for phenotypic memory T cell markers by flow cytometry. To ensure exclusion of naive/non-responding cells, only proliferating cells were analyzed. Nearly all responding CD4^+^ and CD8^+^ T cells exhibited either a CM phenotype (CD62L^+^ CD45RA^−^) or effector/EM (CD62L^−^ CD45RA^−^) (Figures 4A,B), independent of the IC expression profile. However, some variation was evident when looking at IC expression on subsets (Figure 4C). We observed a slight increase in CM on PD-1- and TIM-3-expressing CD4^+^ T cells (55.6% ± 1.4 and 55.3% ± 2.4, respectively) compared to LAG-3-expressing CD4^+^ (48.2% ± 4.7). LAG-3- and PD-1-expressing CD8^+^ T cells favored an EM phenotype with less than half the cells exhibiting a CM phenotype (38.1% ± 4.2 and 43.8% ± 1.5, respectively). CD8^+^ T cells expressing TIM-3 had significantly higher frequencies of CM cells compared to the other two IC-expressing CD8^+^ T cell subsets/populations (52.1% ± 0.9). Distribution of memory subsets on IC-positive cells differed between CD4^+^ and CD8^+^ T cells themselves. There were significantly more CD4^+^ T cells bearing a CM phenotype in LAG-3 and PD-1-expressing populations compared to CD8^+^ T cells expressing LAG-3 and PD-1 (Figure 4C). However, CD4^+^ and CD8^+^ T cells that expressed TIM-3 exhibited similar ratios between CM and EM with a slight skewing toward CM (Figure 4C). Overall, these data reflect another qualitative difference between CD4^+^ and CD8^+^ T cells in their IC expression described here in relation to memory phenotype.

## Discussion

Over the last decade, several molecules have been shown to be expressed on T cells isolated from patients suffering from chronic viral infection or tumor burden. Poor immune function has been associated with these T cells, thus classifying them as being “exhausted.” Clinical studies evaluating blockade of individual IC molecules, such as PD-1, have shown promising results. In one trial, a response rate of 20–25% was reported for patients including those with renal cell carcinoma, melanoma, non-small cell lung carcinoma, and prostate cancer (19). In a separate clinical trial, a 38% response rate was reported for patients with advanced melanoma after treatment with an anti-PD-1 antibody (20). As a means to further increase the response rate, a logical next step is the exploration of potential combinatorial treatments that target multiple IC. To date, concurrent targeting of CTLA-4 and PD-1 in the clinic with antagonist antibodies exhibited slightly enhanced therapeutic efficacy compared to mono-therapy with either agent (22), thus encouraging investigation of additional combination treatments involving other IC molecules. Indeed, a recent study showed that in a preclinical model, resistance to anti-PD-1 treatment could be overcome by blockade to additional IC, such as TIM-3. The authors also reported that patients who became resistant to anti-PD-1 treatment exhibited upregulation of TIM-3 and other IC (23).

Targeting of IC may lead to pleiotropic effects due to the diversity of cell populations that express these molecules. While expression of PD-1 and TIM-3 on CD4^+^ and CD8^+^ T cells has been reported, it has mainly been in the context of being a phenotypic indicator of T cell exhaustion (17, 24, 25); in the context of *in vitro* allogeneic T cell priming, the expression of TIM-3 and PD-1 is not uniformly high. One possible explanation for this is that these two molecules are reciprocally expressed, meaning that they share some mechanism that regulates their expression ensuring that when PD-1 is upregulated TIM-3 is not, and *vice versa*. This mechanism of regulation is feasible but would not necessarily apply directly following priming. As shown in Figure 2, PD-1 is expressed equivalently on CD4^+^ and CD8^+^ T cells for the first few rounds of division, which correlates with the time period immediately following antigen encounter. During this same window of time, the TIM-3 expression profile already differs between CD4^+^ and CD8^+^ T cells, indicating that very early after antigen encounter, both PD-1 and TIM-3 are upregulated to a greater extent on CD8^+^ T cells. Based on the data shown herein, both CD4^+^ and CD8^+^ T cells immediately upregulate PD-1 expression, but in contrast to CD8^+^ T cells, CD4^+^ T cells maintain lower TIM-3 expression levels while increasing PD-1. This may be in response to soluble factors being released during the MLR, as it has been recently shown that γc cytokines can differentially regulate TIM-3 and PD-1 expression on HIV-specific T cells (26). Future studies would include testing the impact of blocking individual γc cytokines (i.e., IL-2, IL-7, IL-15, and IL-21) in the MLR on IC expression. Another possible explanation is that TIM-3 and PD-1 are independently regulated, but have different expression kinetics. This could suggest that TIM-3 and PD-1 mediate non-overlapping functions; though PD-1 is well documented to be a negative regulator in T cell function, TIM-3 may play a different role not limited to downmodulation of T cell activity. Note that cytokine production and proliferation continue throughout the culture period, suggesting T cell functionality is maintained. While these studies examined a limited cohort of samples (*n* = 4), the data suggest that activated CD4^+^ T cells exhibit higher PD-1 expression compared to CD8^+^ T cells with comparably lower levels of TIM-3 expression, while the inverse expression pattern is observed on activated CD8^+^ T cells (i.e., higher TIM-3 and lower PD-1). Although the functional implications of these findings are beyond the scope of this report, results published by others have been mixed regarding expression of IC and T cell function. One study has shown that, in the context of Hepatitis B infection, inhibitory CD4^+^ T cells maintained high PD-1 expression with low TIM-3 (27). However, another group claimed that increased numbers of CD4^+^ PD-1^high^TIM-3^negative^ T cells in follicular lymphoma patients did not correlate to clinical outcome and that these cells were in fact still functional. In the same study, CD4^+^ PD-1^low^TIM-3^positive^ T cells displayed reduced cytokine production and signal transduction (28). While we did not observe differences in the level of cytokine production between IC-expressing subsets, our data indicate that cytokine-producing CD8^+^ T cells are higher in TIM-3 expression. However, this observation was made in the context of an acute primary immune response and does not reflect the conditions of chronic antigen stimulation. In another report, it has shown that virus-specific CD8^+^ T cells expressing high TIM-3 were, in fact, less functional compared to TIM-3^low^ CD8^+^ T cells (29).

Following an acute infection and subsequent clearing of the pathogen, T cells enter a contraction phase in which the majority of the cells undergo apoptosis, while a small subset become memory T cells that are able to persist long term even in the absence of further antigen stimulation. Upon re-encounter with cognate antigen, memory T cells quickly re-activate, proliferate, and acquire effector functions (30). It has been shown that expression of PD-1, TIM-3, and LAG-3 is predominantly associated with an EM phenotype on CD8^+^ T cells (13, 14, 31); however, such information regarding CD4^+^ memory T cells remains insufficient. In the studies reported herein, LAG-3, PD-1, and TIM-3 expressing CD4^+^ and CD8^+^ T cells encompass both EM and CM subsets as classified here by CD62L expression, a marker generally associated with CM when expressed on CD45RA^−^ T cells (32). Using these criteria, PD-1, TIM-3, and LAG-3 were detectable on both EM and CM T cells. PD-1 and LAG-3 expression were associated with EM phenotype at a slightly higher frequency on CD8^+^ T cells. However, TIM-3 expression was more balanced between CM and EM populations for both CD4^+^ and CD8^+^ T cells. We acknowledge these as preliminary phenotypic observations, and more in-depth analyses involving additional memory markers, including but not limited to CCR7 and CD95, are required to gain a more complete definition of the cells. It is possible that the frequency of CM and effector/EM cells observed here constitute only a snapshot of the differentiation process occurring in a primary immune response (33). However, another possibility is that our MLR conditions are expanding existing cell populations of a particular memory phenotype (i.e., central or effector), rather than inducing differentiation. The higher CD62L expression observed on all IC-expressing CD4^+^ T cells may reflect a different stage in differentiation than CD8^+^ T cells. Alternatively, it has also been reported that CD4^+^ CD62L^+^ CM T cells can be converted to FoxP3^+^ T cells and that this may be a regulatory mechanism to control proliferation of effector T cells (34). In the context of the MLR, it is possible that IC-expressing CD4^+^ CM cells, following activation, can convert to regulatory T cells to control activated alloreactive CD8^+^ T cells. Further studies are necessary to discriminate these mechanisms and understand the potential role that IC plays in T cell differentiation, and *vice versa*, as well as immune regulation.

Downregulation of PD-1 on CD8^+^ T cells is associated with acute liver allograft rejection (35). In addition, negative signaling by TIM-3-expressing CD4^+^ T cells has been shown to confer protection of allogeneic liver transplants in mice (36, 37). An elegant and comprehensive review by McGrath and Najafian (38) outlines the roles of IC signaling in tolerance and applications targeting IC in transplantation, some already in advanced clinical trials (i.e., CTLA-4-Ig). There have been several groups exploring the use of other fusion proteins, including PD-L1-Ig and B7-H4-Ig, in preclinical allograft models. The use of agonistic IC mAb (i.e., anti-BTLA) and stable IC ligands (i.e., galectin-9) has also shown promising results in various transplant models. However, there is still much to be understood before any of these IC therapeutics can be applied in the clinic.

Overall, our *in vitro* findings show that there are differences in expression of IC molecules between CD4^+^ and CD8^+^ T cells and suggest that further studies are needed to understand the functional significance of IC expression patterns between CD4^+^ and CD8^+^ T cells. Therapeutic targeting of IC molecules becomes a challenge with mixed cell populations due to broad expression patterns of some IC molecules on other cell types. For example, TIM-3 is known to be expressed on innate cells, including DCs, macrophages, and NK cells (39–42); thus, TIM-3 blockade likely would not solely target exhausted T cells. Expression of IC on cell types other than T cells would act as potential “sinks” for any therapeutic targeting of these molecules, resulting in dosing inconsistencies as well as potential unexpected on-target (but off-T-cell) effects on other IC-expressing cell populations. Given the diverse distribution of IC expression on T cells and other cell types, one way to enhance cell-specific IC targeting would be to include a T cell-specific, or even CD4^+^ or CD8^+^ specific, moiety in a therapeutic, thereby increasing the likelihood of binding to IC on a particular cell type. Likewise, if the intent was to target non-T cells, alternate cell-specific (e.g., CD11c) targeting domains could be included in a therapeutic. Another strategy would be to administer different IC blockade (or agonist) to patients depending on their disease state, since kinetics of the immune response can drive upregulation or downregulation of various IC on different cell types. Further studies with different donors (healthy and diseased) could contribute to a more comprehensive understanding of expression patterns and kinetics, which is undoubtedly required for optimal execution of therapeutics targeting IC. Differences in the kinetics and expression levels of these markers must be taken under careful consideration when designing drugs and biologics aimed to target specific ICs as they may favor certain T cell subsets over another, leading to a wide range of therapeutic outcomes.

## Author Contributions

NS contributed to data generation and analysis and prepared the manuscript. BH, SS, and LB contributed to data generation and analysis. SS-M contributed to data generation and analysis and reviewed the manuscript.

## Conflict of Interest Statement

The authors declare that the research was conducted in the absence of any commercial or financial relationships that could be construed as a potential conflict of interest.
