# Supplementary material for: Differential Expression of Immune Checkpoint Modulators on In Vitro Primed CD4+ and CD8+ T Cells
Source: Front Immunol. 2016 Jun 16;7:221. doi: 10.3389/fimmu.2016.00221 (PMC4909735; doi:10.3389/fimmu.2016.00221)
Supplement: Supplementary file 1 [file Presentation_1.PDF]

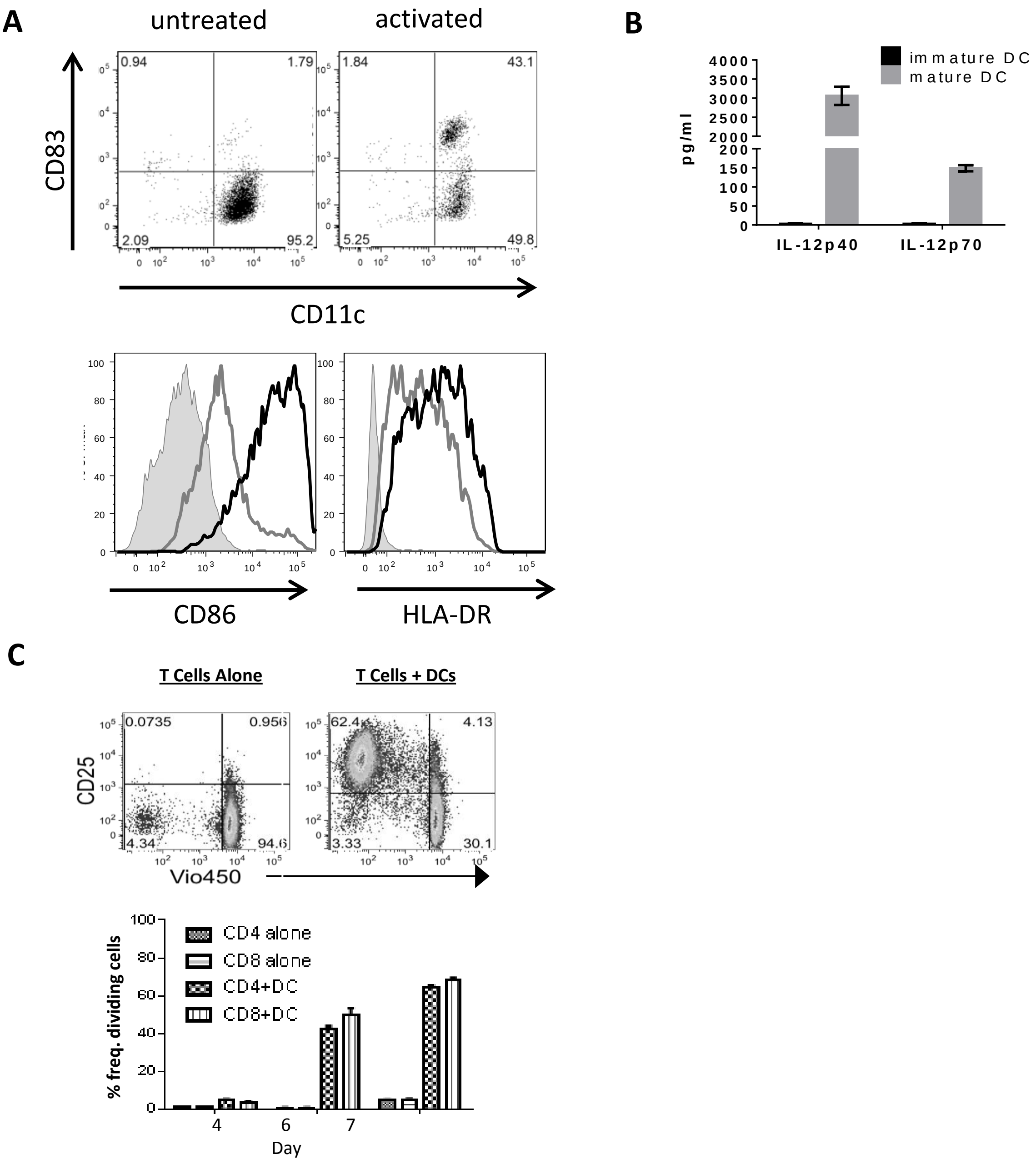

Supplemental Figure 1. Stimulation by activated allogeneic DC induces T cell activation. Human DC were activated with IFN-γ and LPS. **(A)** Representative flow cytometric dot plots depicting expression of CD11c and CD83 (upper panels), and histograms showing CD86 and HLA-DR on activated DC (black line) compared to untreated DC (gray line) and isotype control (shaded) (lower panels). **(B)** Supernatants collected from untreated DC (gray) and activated DC (black) and analyzed for IL-12p40 and IL-12p70 production by ELISA. **(C)** T cell proliferation as measured by dye dilution and CD25 expression when cultured alone or with DC for 7 days (top panel) and time-course for percent frequency of dividing cells based on proliferation dye dilution, gated on CD4+ or CD8+ T cells (bottom panel). Data are representative of four different experiments.

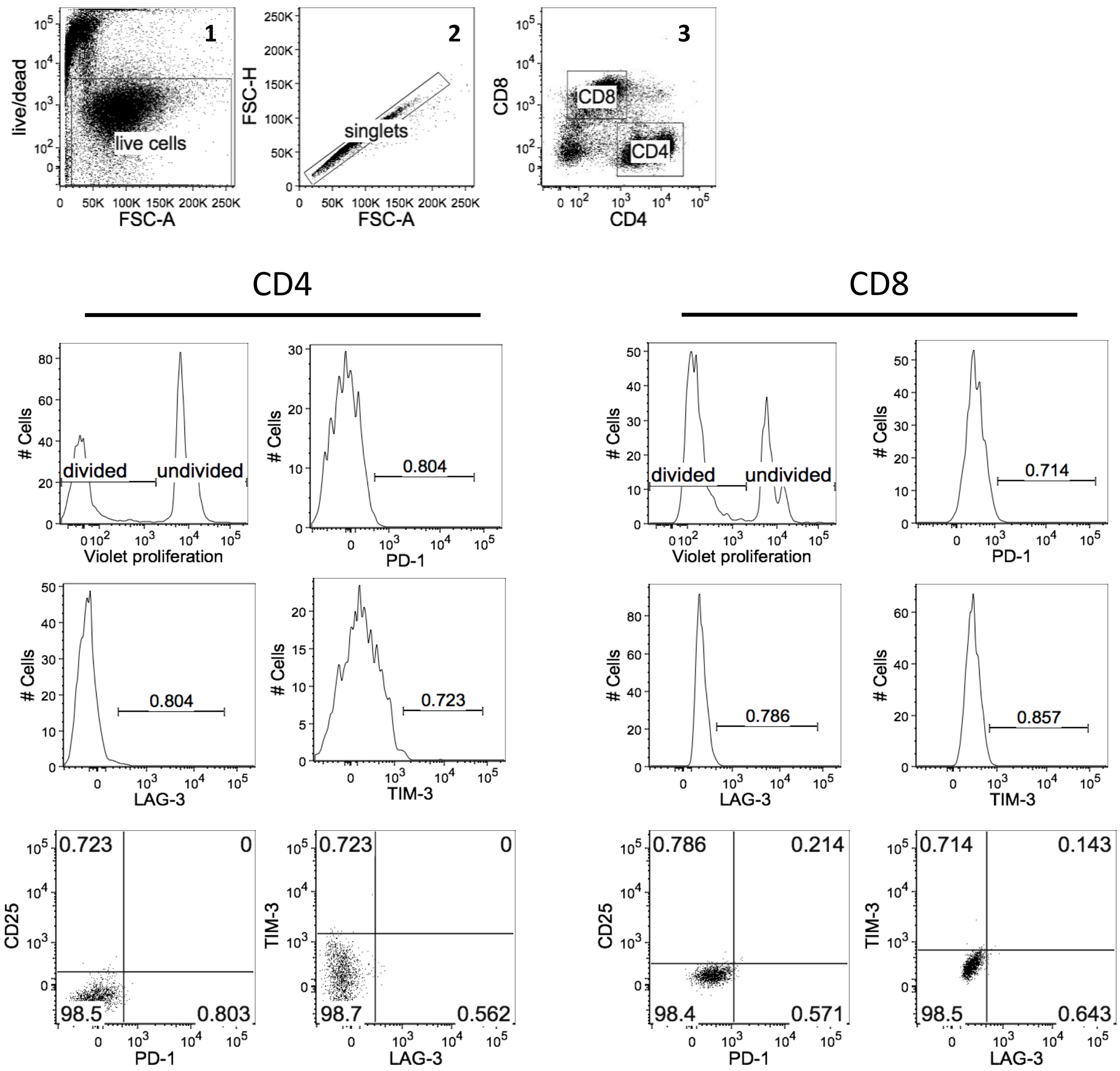

Supplemental Figure 2. Complete gating strategy for flow cytometry data. Representative flow cytomeric dot plots and histograms showing gating strategy for T cell analyses by (1) excluding dead cells, (2) excluding doublets, (3) gating on CD4+ or CD8+ T cells, and then gating on dividing or non-dividing cells for CD4 (left panels) and CD8 (right panels). Gates were established to include 0.5-1% positve events based on isotype control staining of CD4+ and CD8+ T cells. These gates were then applied to experimental staining samples to determine single and double positive expression of IC on CD4+ and CD8+ T cells.
